# Micro-RNA 122 and micro-RNA 96 affected human osteosarcoma biological behavior and associated with prognosis of patients with osteosarcoma

**DOI:** 10.1042/BSR20201529

**Published:** 2020-12-14

**Authors:** Bin Liu, Shuqiang Yao, Jiping Zhou

**Affiliations:** Department of Orthopedic Surgery, Shandong Wendeng Osteopathic Hospital, Weihai, Shandong, China

**Keywords:** miRNA-122, miRNA-96, prognosis, osteosarcoma

## Abstract

Osteosarcoma (OS) is the most common bone malignancy in both children and adolescents. In the present study, we aimed to explore the association of miRNA-122 and miRNA-96 expression with the clinical characteristics and prognosis of patients with osteosarcoma. The expression of miRNA-122 and miRNA-96 in human osteosarcoma cell lines and tissues were detected in the present study. Reverse transcriptase-PCR (RT-PCR) was used to determine the expression levels of miRNA-122 and miRNA-96 in 68 human OS samples. We found that MiRNA-122 and miRNA-96 were widely up-regulated in osteosarcoma, gastric cancer and pancreatic cancer. In HOS, Saos-2 and U2OS osteosarcoma cells, miRNA-122 and miRNA-96 were up-regulated significantly, while down-regulated in MG-63 cells. After further investigation, we found that miRNA-122 and miRNA-96 concentrations were significantly higher in the tumor tissues than those in the normal tissues (*P*<0.01). Moreover, the cell proliferation of LV-miRNA-122-RNAi and LV-miRNA-96-RNAi transfected SaOS2 was significantly decreased compared with the LV- miRNA-122-RNAi-CN and LV- miRNA-96-RNAi group. After adjusting for competing risk factors, we found combined high miRNA-122 and miRNA-96 expression was identified as independent predictor of overall survival.

## Introduction

Osteosarcoma is a malignant tumor that cells occur in skeleton and affiliates, which has been reported to present aberrant growth and migration in osseous tissues [1,2]. Osteosarcoma is the most common bone malignancy in children and adolescents and may lead to the possibility of other malignancy [3]. In recent years, new strategies have been proposed and suggested to improve the overall survival for patients with osteosarcoma [4,5]. Major advances have been proposed for the treatment of osteosarcoma with the discovery of several chemotherapeutic and immunologic agents [6]. However, the overall survival remains with little improvement since the introduction of neoadjuvant chemotherapy, radiotherapy and surgery.

MiRNA dysregulation is likely to occur in all the stages of carcinogenesis [7–9]. Moreover, miRNA profiles can differentiate between healthy people and those affected with cancers. Such profiles are also different in benign and malignant tissues and they may be different in terms of malignancy by their sub-type. MiRNAs can be diagnosed in tumors, serums, plasmas and urines that can be considered as a noninvasive method for evaluating responses toward treatment [10–12]. Along with the advance in high-throughput screening and bioinformatics, a growing number of important regulatory microRNAs in cancer have been discovered. A discussion on the biological functions of differentially expressed microRNAs in osteosarcoma can bring new hope for diagnosis and treatment. The present study first identified the differentially expressed microRNAs using microRNAs expression microarray. Then, miRNA-122 and miRNA-96 were selected by bioinformatics technique, followed by RT-PCR in tumor samples and cell lines.

MiRNA-122 is located in the intragenic region of 18q21.31. According to the significant role of miRNA-122 in carcinogenesis, its expression can be biomarker for prognosis predicting of patients with cancers [13–15]. The recent studies have indicated that miRNA-122 can have a potential to be used as a blood marker in hepatic diseases. Researchers showed that a serum level of miRNA-122 was able to differentiate between healthy people and HBV-infected ones. Some of the circulating miRNA changes also happened due to the severity of HBV symptoms and miRNA-122 expression in these patients could be reported significantly higher than that in healthy individuals [16,17]. As for miRNA-96, studies revealed that its expression was higher in multiple sclerosis (MS) patients in remission than in relapse as well as in controls, and was significantly higher in controls than in MS patients in relapse [18]. They concluded that miRNA-96 might be characteristic of the remitting phase of the disease. The genes targeted by miRNA-96 are thought to be involved in immunological pathways such as interleukin signaling [19,20].

Many miRNAs have been studied in osteosarcoma and we have experiences in sorting significant miRNAs for predicting both diagnosis and prognosis. specifically, the present study aimed to investigate the prognostic value of plasma micro-RNA 122 and micro-RNA 96 in patients with osteosarcoma.

## Patients and materials

### Cell culture

Human osteosarcoma cell lines (HOS, Saos-2, U2OS and MG-63) and normal osteoblast cells (NHOst) were obtained from the Chinese Cell Bank of the Chinese Academy of Sciences (Beijing, China). All cells and were cultured in DMEM medium supplemented with 10% heat-inactivated fetal bovine serum (FBS), 100 U/ml of penicillin and 100 μg/ml of streptomycin. They were all placed in a humidified atmosphere containing 5% CO_2_ at 37°C.

### CCK8 assay

Cell growth was measured using the cell proliferation reagent WST-8 (Roche Biochemicals, Mannheim, Germany). After plating cells in 96-well microtiter plates (Corning Costar, Corning, NY) at 1 × 10^3^/well, 10 μl of CCK8 was added to each well at the time of harvest, according to the manufacturer’s instructions. One hour after adding CCK8, cellular viability was determined by measuring the absorbance of the converted dye at 450 nm.

### Wound healing assay

The cells were cultured on six-well plates with DMEM medium containing 10% FBS. When the cell density reached 70–80%, the bottom of the plate was scratched with a 100 μl pipette tip to create a cell-free gap, following which the cells were incubated for 48 h with DMEM medium containing 1% FBS. An inverted Olympus IX50 microscope (Olympus Corp., Tokyo, Japan) was used to obtain phase-contrast images of the wound healing process at different time points after scratching. The size of the healed wound was then compared with the size of the initial wound.

### Transwell assay

A total of 5 × 10^4^ cells suspended in 100 μl serum-free DMEM medium were seeded into the upper chamber of a Transwell apparatus (Corning Inc., Corning, NY, U.S.A.; 8 μm pore) with 50 μl Matrigel (BD Biosciences, Franklin Lakes, NJ, U.S.A). A total of 600 μl DMEM medium containing 10% FBS was added to the lower chamber. Following incubation at 37°C for 48 h, cells in the upper chamber were removed with a cotton swab and cells on the lower surface were fixed in 1% paraformaldehyde followed by staining with 0.1% Crystal Violet solution (Beyotime Institute of Biotechnology, Haimen, China) at room temperature. The number of invading cells was determined for five randomly selected fields (×200 magnificatoin) under a microscope (Leica inverted microscope DMi1; Leica, Wetzlar, Germany). Three independent experiments were performed and the mean was calculated.

### Patients, osteosarcoma specimen

About 68 patients (age range: 8–50 years, median 17.2 years) with osteosarcomas tissues and corresponding noncancerous bone tissue samples from the same specimens were collected from Department of Orthopedic surgery, Shandong Wendeng Osteopathic Hospital between July 30, 2010 to May 1 2018. All patients were treated with preoperative chemotherapy lasting for 4 months, using either the combination of an anthracycline (doxorubicin) and high-dose methotrexate or the combination of etoposide, ifosfamide and high-dose methotrexate. The present study was approved by the Institutional Review Board of Shandong Wendeng Osteopathic Hospital and the IRB number is WD20135921. All patients were given written informed consent to participate. The data did not contain any information that could identify the patients. Patients were excluded from this study if they had a history of other solid tumors, or died from severe postoperative complications. The clinicopathological information of all patients was shown in Table 1.

**Table 1:** Patient and tumor characteristics

Variable	Patients with osteosarcoma
**No.**	68
**Age in years**	
≤20	35
>20 and <40	20
>40	13
**Sex**	
Female	25
Male	43
**Tumor location**	
Femur	36
Shin	20
Others	12
**Enneking**	
IIIB	32
III	36
**Tumour characteristics**	
**Histology (main component)**	
Conventional osteosarcoma	46
Telangiectatic osteosarcoma	10
Parosteal osteosarcoma	4
Small round cell osteosarcoma	5
Fibrous tissue osteosarcoma	3
**Surgical**	
Salvage	14
Amputation	54
Expression of miRNA-122	
High	36
Low	32
**Expression of miRNA-96**	
High	31
Low	37

### Total RNA extraction, cDNA synthesis and reverse transcription PCR, and quantitative

The total RNA was extracted from SaOS2 cell cultures using RNA Extraction Kit (Tiangen, Beijing, China), according to the manufacturer’s instruction. Complementary DNAs (cDNAs) were synthesized by a reverse transcription kit (Invitrogen, CA, U.S.A.). The detailed protocol of the RNA extraction and cDNA synthesis were performed according to previous reports. Quantitative real-time reverse transcription-PCR (RT- PCR) was carried out using the SYBR Premix Ex TaqTM II (TaKaRa) according to the manufacturer’s protocol. The real-time PCR protocol was as follows: stage 1: denaturation at 95°C for 30 s; stage 2: 40 repeats of 95°C for 5 s and 60°C for 30 s; and stage 3: dissociation. Both the reactions and relative levels of the target genes were quantified by C1000TM Thermal cycler (Bio-Rad CFX96TM Real-time System).

### Silencing

Small interfering RNA (siRNA) targeted to human miRNA-122 and miRNA-96 were designed following the procedure described by Reynolds et al. Selected siRNAs were inserted into the Lentiviral vector (Ambion, Austin, TX, U.S.A.). Once we obtained Lv-miRNA-122-siRNAs and miRNA-96-siRNAs targeting the related mi-RNAs and Lv-mi-RNA-siRNA-CON, SaOS2 cell cultures were transduced at varying multiplicities of infection (MOIs) to obtain a considerable silencing effect.

### Statistical analysis

Statistical Package for the Social Sciences (SPSS) version 16.0 program was used for statistical analysis. In the evaluation of study data, in addition to descriptive statistical methods (mean, standard deviation, median, frequency, rate, minimum and maximum), Student’s *t-*test was used for comparing variables showing normal distribution and Mann–Whitney *U* test for comparing variables not showing normal distribution within qualitative data. Pearson’s Chi-Square test, Fisher’s Exact Test and Yates Continuity Correction Test were used for comparing qualitative data. Kaplan–Meier survival analysis and log-rank were used for evaluating survival. Statistical significance was evaluated at the level of *P*<0.05.

## Results

### Patients’ characteristics

About 68 patients with osteosarcoma were recruited into the present study. Of the all the patients, the median follow-up was 3.8 years (range: 7.8 months to 5.9 years). The baseline characteristics of patients at diagnosis were summarized in Table 1.

### Gene expression detected by RT-PCR in each cell line

**Figure 1:**
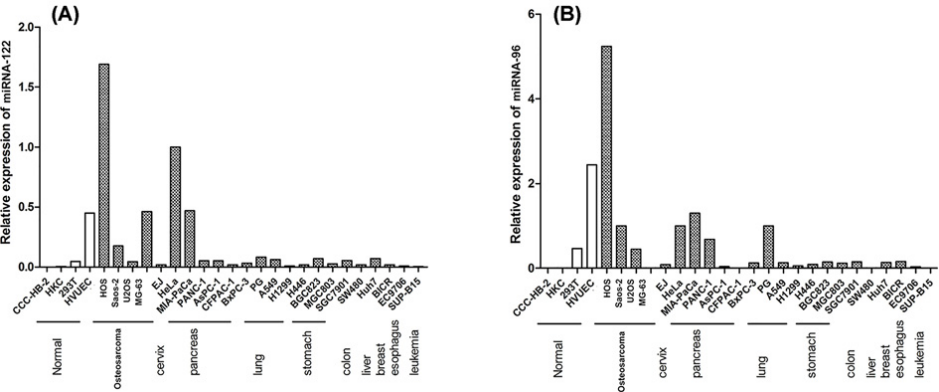
Detections of the targeted microRNAs (miRNA-122 and miRNA-96) in different cancer cell lines (**A**) miRNA-122 expression in various cancers; (**B**) miRNA-96 expression in various cancers.

We performed further detections of the targeted microRNAs in different cancer cell lines. Mi-RNA-122 and miRNA-96 were not expressed in the normal cell lines CCC-HB-2 and HKC, down-regulated in 293T cells and up-regulated in HUVECs. MiRNA-122 and miRNA-96 were widely up-regulated in osteosarcoma, gastric cancer and pancreatic cancer. In HOS, Saos-2 and U2OS osteosarcoma cells, miRNA-122 and miRNA-96 were up-regulated significantly, but down-regulated in MG-63 cells. For example, in HOS cells, miRNA-122 expression was higher by 1.69 times (*P*<0.05) as compared with the HeLa cells, 24705.00 times compared with CCC-HB-2; while miRNA-96 expression was higher by 5.24 times compared with the HeLa cells, 4471.00 times compared with CCC-HB-2 (*P*<0.05; Figures 1A and 1B).

### LV- miRNA-122-RNAi and LV- miRNA-96-RNAi inhibit the proliferation of cells

**Figure 2:**
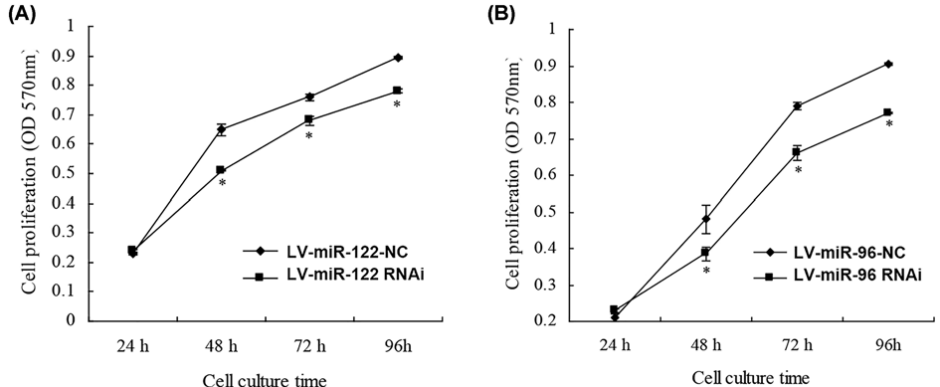
MTT analysis for cell lines (**A**) LV- miRNA-122-RNAi and (**B**) LV- miRNA-96-RNAi inhibit the proliferation of cells. *P<0.05.

The CCK8 assay results indicated that the cell proliferation of LV- miRNA-122-RNAi and LV- miRNA-96-RNAi transfected SaOS2 were significantly decreased compared with the LV- miRNA-122-RNAi-CN and LV- miRNA-96-RNAi groups (Figure 2A,B; *P*<0.05). These results suggest that the miRNA-122 and miRNA-96 may participate in the proliferation of the human osteosarcoma cells.

### LV- miRNA-122-RNAi and miRNA-96-RNAi inhibit SaOS2 cells invasion and migration *in vitro*

**Figure 3:**
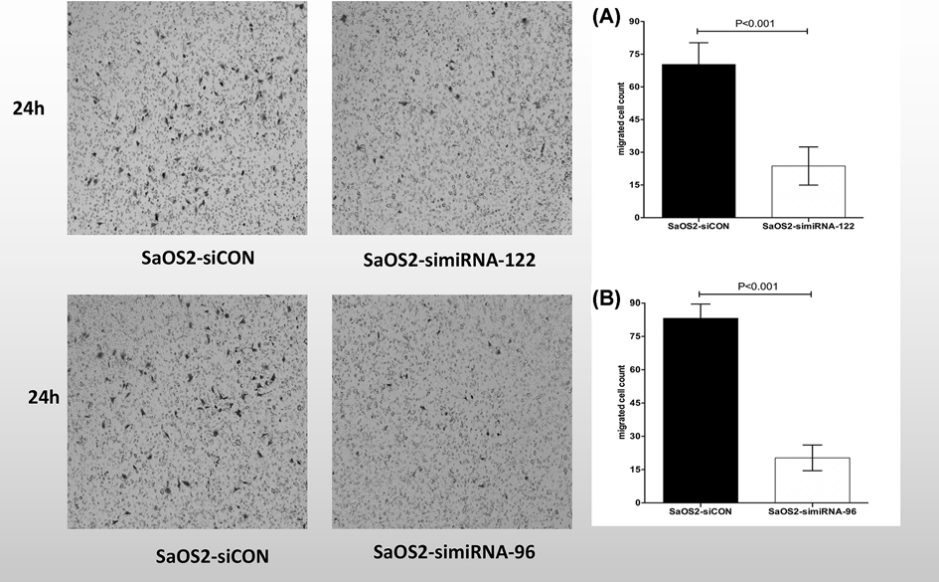
Effect of LV-miRNA-122-RNAi and miRNA-96-RNAi on the invasion ability of SaOS2 cells Transwell experiments showed that SaOS2 cell invasion and staining were treated with both miRNA-122-RNAi and miRNA-96-RNAi 24 h after transfection; the quantitative results of membrane transfection ability of different groups of cells can be seen in both (**A**) and (**B**), *P*<0.001 represents that the invasive ability of SaOS2 cells was significantly inhibited in the transfected LV- miRNA-122-RNAi and miRNA-96-RNAi group compared with the blank transfection group.

**Figure 4:**
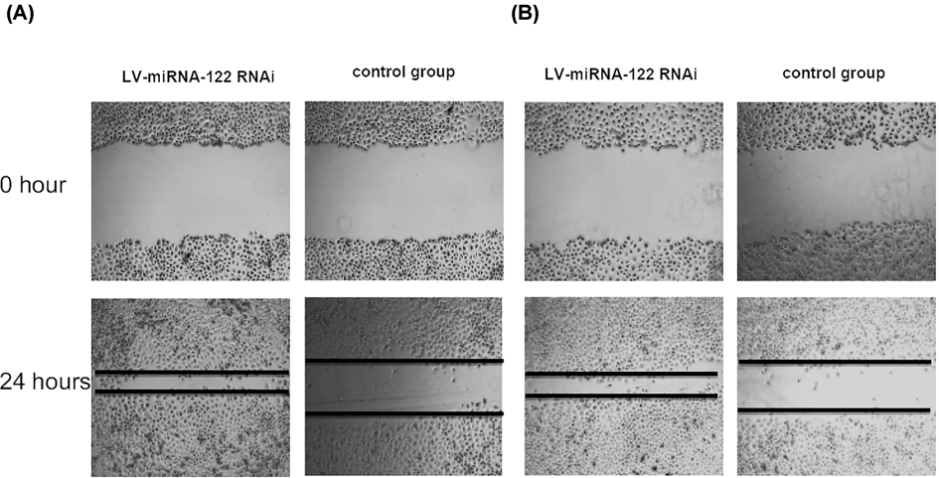
Effect of LV-miRNA-122-RNAi and miRNA-96-RNAi on the migration ability of SaOS2 cells Cell scratch experiments showed that the migration and staining of SaOS2 were treated with LV-miRNA-122-RNAi and miRNA-96-RNAi 24 h after transfection; the quantitative results of the transmembrane ability of different grouped cells can be seen in both (**A**) and (**B**).

We tested the effects of LV-miRNA-122-RNAi and miRNA-96-RNAi on the invasion ability of SaOS2 cells through scratch experiments and Matrigel invasion experiments. The results showed that compared with the blank LV-RNAi-CN transfection group, the invasion ability of SaOS2 cells in the LV-miRNA-122-RNAi and miRNA-96-RNAi transfection group were significantly inhibited (Figure 3A,B; *P*<0.001). Moreover, the effect of LV-miRNA-122-RNAi and miRNA-96-RNAi on the migration ability of SaOS2 cells was also tested by scratch. Similar to the results of cell invasion experiments, compared with the blank LV- RNAi-CN transfection group, the migration ability of SaOS2 cells in the LV-miRNA-122-RNAi and miRNA-96-RNAi transfection group was significantly inhibited (Figure 4A,B; *P*<0.01). Based on the above results, we believe that transfection of LV-miRNA-122-RNAi and miRNA-96-RNAi can inhibit the invasion and migration ability of SaOS2 cells *in vitro*.

### Comparing of plasmatic miRNA-122 and miRNA-96 expression in osteosarcoma and normal tissues patients

**Figure 5:**
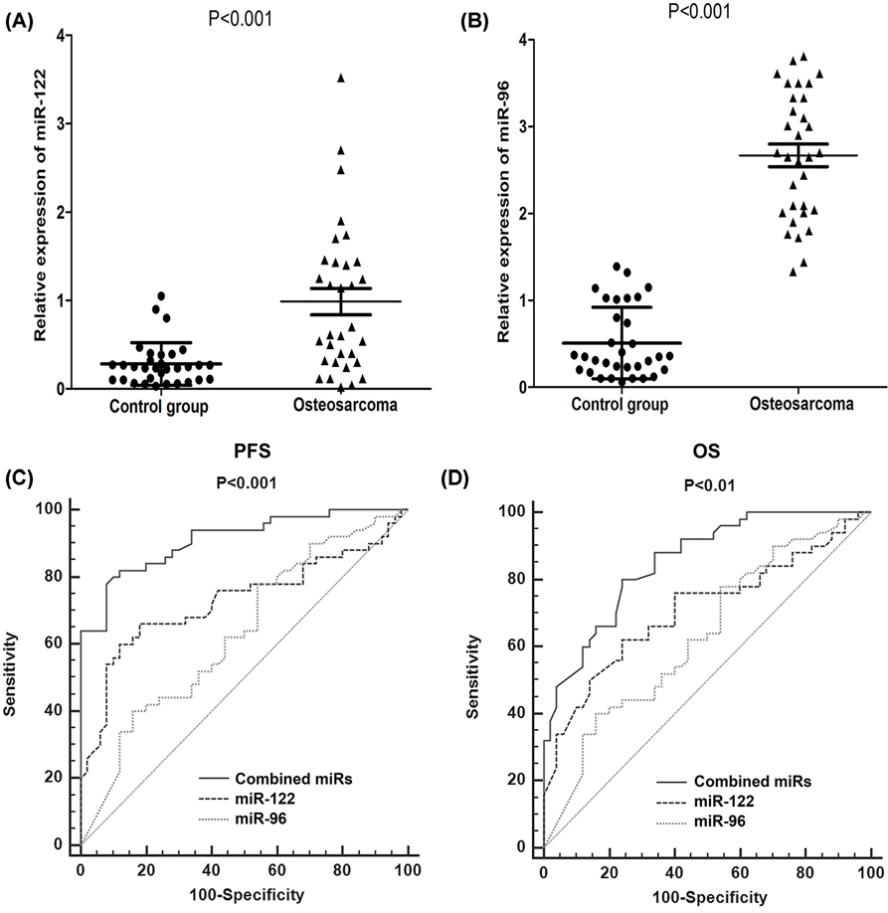
Predictive values of mi-RNAs in patients (**A** and **B**) miRNA-122 and miRNA-96 concentration were significantly higher in the tumor tissues than in the normal tissues (*P*<0.01). (**C** and **D**) The AUC of combined miRNA-122 and miRNA-96 was larger than solely miRNA-122 and miRNA-96 in predicting PFS and OS.

MiRNA-122 and miRNA-96 expression were examined by qRT-PCR. Notably, miRNA-122 and miRNA-96 concentration was significantly higher in the tumor tissues than in the normal tissues (*P*<0.01, Figure 5A,B). These results were consistent with the detection of miRNA-122 and miRNA-96 in osteosarcoma cell lines.

### Combined miRNA-122 and miRNA-96 overexpression was an independent predictor of OS for patients with osteosarcoma

Cox proportional hazards models were then used to quantify the prognostic significance of risk factors after multivariable adjustment. A multivariable analysis was performed to assess the factors that demonstrated significant effects as in the univariate analysis. After adjusting for competing risk factors, combined miRNA-122 and miRNA-96 expression and tumor location remained independent predictors of OS. The details are shown in Table 2.

**Table 2:** Cox proportional hazard regression analyses in the osteosarcoma

Variable	HR	Univariate 95%CI	*P* value	HR	Multivariate 95%CI	*P* value
**Age in years**	1.021	0.942–1.293	0.489			
**Male**	1.034	0.932–1.539	0.405			
**Tumor location**	1.651	1.295–2.402	<0.001	1.573	0.135–2.529	0.023
**Enneking**	1.129	0.903–1.302	0.250			
**Histology (main component)**	1.185	0.949–1.761	0.072			
**Surgical**	1.358	1.255–1.952	0.015	1.103	0.912–1.493	0.293
**Expression of miRNA-122**	1.577	1.221–2.346	0.002	1.049	0.842–1.294	0.392
**Expression of miRNA-96**	1.852	1.421–2.635	<0.001	1.156	0.901–1.458	0.241
**Combined miRNA-122 and miRNA-96**	2.071	1.511–3.035	<0.001	1.434	1.257–2.766	0.021

Abbreviation: CI, confidence interval.

### Comparing of prognostic performance for osteosarcoma patients among the expression of miRNA-122 and miRNA-96 levels

For predicting of prognosis in patients with osteosarcoma, combined miRNA-122 and miRNA-96 expression had significant superior predictability compared with solely miRNA-122 and miRNA-96. The AUC of combined miRNA-122 and miRNA-96 was 0.829 (95% CI: 0.764–0.887), which was larger than solely miRNA-122 (0.675, 95% CI: 0.547–0.787) and miRNA-96 (0.698, 95% CI: 0.552–0.763) in predicting PFS (Figure 5C). The AUC of combined miRNA-122 and miRNA-96 was 0.861 (95% CI: 0.752–0.935), which was larger than solely miRNA-122 (0.701, 95% CI: 0.562–0.802) and miRNA-96 (0.698, 95% CI: 0.552–0.763) in predicting OS (Figure 5D).

## Discussion

OS is the most common bone malignancy in children and adolescents, and it comprises approximately 3% of all pediatric tumors [21]. Despite aggressive multi-modality therapy applied, patients with advanced disease still have a poor prognosis with a 5-year survival rate at only 10–20% [22]. The poor prognosis of OS is associated with tumor invasion and metastasis, which often lead to therapeutic failure. miRNAs are short (20–23 nucleotides) single-stranded noncoding RNAs that play an important role in posttranscriptional regulation of gene expressions [23–26]. These molecules have key roles in biological processes and are involved in the pathogenesis of several diseases. Several studies have shown that miRNAs play therapeutic roles in various diseases such as cancer, digest diseases and stroke [27–29]. These studies confirmed that antagomirs (anti-miRNAs) can serve as an effective treatment in enhanced cell survival in various animal models. Some reports observed that some miRNAs show therapeutic effects and be identified as target biomarkers [30–32]. As been reported, CCNG1 is the target of miRNA-122 and an inverse relation exists between them in HCC-derived cell lines and HCC tissues. CCNG1 is the only known cyclin that bears two functional binding sites for p53 tumor suppressor protein and is transcriptionally triggered by this transcription factor [33]. MiRNA-122 was shown to increase p53 protein levels and activity through its negative regulation of cyclin G1 [34]. Specifically, miRNA-122 has a high expression in the liver and tumor suppressor-like qualities and evidence suggests that miRNA-122 is necessary for HCV replication and hepatocyte differentiation and homeostasis. Knockout of the miRNA-122 gene is associated with the loss of the hepatic phenotype and progression to cancer. For miRNA-96, researcher have found the it directly suppressed γ-globin expression and thus contributes to HbF regulation [35].

In present study, we found that miRNA-122 and miRNA-96 were widely up-regulated in osteosarcoma, gastric cancer and pancreatic cancer. In HOS, Saos-2 and U2OS osteosarcoma cells, miRNA-122 and miRNA-96 were up-regulated significantly, but down-regulated in MG-63 cells. After further investigation, Notably, miRNA-122 and miRNA-96 concentration were significantly higher in the tumor tissues than in the normal tissues (*P*<0.01). Moreover, the cell proliferation of LV- miRNA-122-RNAi and LV- miRNA-96-RNAi transfected SaOS2 were significantly decreased compared to the LV- miRNA-122-RNAi-CN and LV- miRNA-96-RNAi group. After adjusting for competing risk factors, combined miRNA-122 and miRNA-96 expression and tumor location was identified as independent predictor of overall survival.

However, there are limitations of the present study: (1) the sample size is small in this study, and further larger sample study is needed to confirm the present experimental results; (2) whether overexpression of miRNA-122 and miRNA-96 have the optimal specificity and sensitivity for osteosarcoma diagnosis and prognosis also needs future confirmation.

In conclusion, we found miRNA-122 and miRNA-96 were widely up-regulated in osteosarcoma cell lines such as HOS, Saos-2 and U2OS. Moreover, miRNA-122 and miRNA-96 concentration was significantly higher in the tumor tissues than in the normal tissues. Combined miRNA-122 and miRNA-96 expression was an independently risk factor associated with the prognosis of patients with osteosarcoma.

## Abbreviations

cDNA: complementary DNA
CI: confidence interval
OS: osteosarcoma
RT-PCR: reverse transcriptase-PCR
